# Epidemiological aspects of mortality and morbidity of dual diagnosis patient

**DOI:** 10.1192/j.eurpsy.2026.10382

**Published:** 2026-07-31

**Authors:** J. G. Bramness

**Affiliations:** Oslo University Hospital, Oslo, Norway

## Abstract

**Abstract:**

Dual diagnosis—co-occurring mental illness and substance use disorder—poses substantial challenges for healthcare systems. Individuals affected by both conditions experience markedly elevated morbidity and mortality compared with patients who suffer from only one disorder. This presentation will clarify key concepts and delineate which conditions should be considered within, and outside of, the dual-diagnosis framework. We will demonstrate that comorbidity is highly prevalent: patients with substance use disorders frequently meet criteria for one or more mental disorders, and conversely, individuals with psychiatric illness often develop substance-related problems.

After outlining definitions, we will present data on prevalence from different populations and countries. Attention will be given to the fact that patients who enter specialist addiction or mental health services tend to represent the more severely affected segment of the population. As a result, clinical samples often show disproportionately high rates of dual-diagnosis conditions, which may unintentionally lead to overestimation of both prevalence and associated health risks when compared with community samples.

We will subsequently highlight morbidity and mortality patterns across several dual-diagnosis constellations, drawing on findings from epidemiological and clinical studies. Towards the end of the lecture, we will focus specifically on substance-induced psychosis, an important and complex subgroup. This condition illustrates the considerable health burden associated with dual diagnosis, including heightened risks for acute complications, chronic illness trajectories, and premature death.

**Disclosure of Interest:**

None Declared

